# Perceived social support and health-related quality of life (HRQoL) in Tehranian adults: Tehran lipid and glucose study

**DOI:** 10.1186/s12955-018-0914-y

**Published:** 2018-05-10

**Authors:** Sara Jalali-Farahani, Parisa Amiri, Mehrdad Karimi, Golnaz Vahedi-Notash, Golshan Amirshekari, Fereidoun Azizi

**Affiliations:** 1 Research Center for Social Determinants of Health, Research Institute for Endocrine Sciences, Shahid Beheshti University of Medical Sciences, P.O.Box: 19395-4763, Tehran, IR Iran; 2 Department of Epidemiology and Biostatistics, School of Public Health, Tehran University of Medical Sciences, Tehran, Iran; 3 Endocrine Research Center, Research Institute for Endocrine Sciences, Shahid Beheshti University of Medical Sciences, Tehran, Iran

**Keywords:** Socio-demographic factors, Social support, Health-related quality of life, Adults, Iran

## Abstract

**Background:**

Several studies have demonstrated the positive association between perceived social support and health-related quality of life (HRQoL) in certain groups; however, few studies have assessed this relationship in general population and between genders. This study aimed to investigate associations between socio-demographic factors, perceived social support and HRQoL among an urban Iranian population.

**Methods:**

The study population were 1036 adults who had participated in Tehran Lipid and Glucose Study (TLGS). Data on socio-demographic information, perceived social support and HRQoL were collected using standard questionnaires by trained interviewers. Perceived social support and HRQoL were assessed using Iranian versions of the Multidimensional Scale of Perceived Social Support (MSPSS) and Short-Form 12-Item Health Survey version 2 (SF-12v2) respectively. Data on sets of associations among socio-demographic factors, perceived social support and quality of life were analyzed using Structural Equation Modeling (SEM) with IBM SPSS AMOS software.

**Results:**

Mean ages were 50.3 ± 16.3 and 49.6 ± 14.0 years in men and women respectively and 40.9% of participants were male. In terms of perceived social support scores, except for family subscale scores (*p* = 0.003), there were no significant differences between men and women. However, men had significantly higher HRQoL scores, compared to women in all subscales. The findings of SEM analysis demonstrated that being married in both genders (*p* < 0.001) and lower age in men (*p* < 0.05) were significantly associated with higher level of perceived social support. In terms of physical HRQoL, being single and higher perceived social support in both genders and lower age and not having any chronic diseases, only in women were associated with higher physical HRQoL. However, for mental HRQoL, age and perceived social support had significant direct associations with mental HRQoL in both genders (*p* < 0.001); in women, being single (*p* < 0.05) and not having chronic diseases (*p* < 0.001) were also significantly associated with better mental HRQoL.

**Conclusion:**

Perceived social support was found to be both directly and indirectly associated with physical and mental aspects of HRQoL in both genders. Current structural models provide beneficial information for planning health promotion programs aimed at improving HRQoL among Tehranian adults.

## Background

Following the sweeping changes worldwide in the pattern of illnesses and the rising prevalence of non-communicable diseases (NCDs) over the past decades, the medical framework has been changed and besides life expectancy, quality of life (QOL) has become critically important [1, 2]. Accordingly, beyond measurable objective outcomes, such as mortality and clinical functions, improving health-related quality of life (HRQoL) as an individuals’ self-evaluation of physical, mental, and social health status based on their experiences and perceptions, is now the ultimate goal of disease prevention programs being considered at different levels of health care [3–5].

Different demographic, psychological, environmental and social relations and conditions are known to be associated with HRQoL by data available in general populations and those with specific diseases in different stages of life [6–12]. Among these factors, findings regarding the influence of social support on individuals’ disease recovery, coping resources and HRQoL are remarkable [7, 8, 13–17]. Social support as a multidimensional construct encompasses the kind of interpersonal interaction and relationship, individual’s belief that he/she is cared for and loved, esteemed and valued, and is a part of the communication network [18–20]. Two main aspects of received and perceived social support have been considered in current literature; while received social support implies the particular supportive behavior which is provided to recipients by their supportive networks, perceived social support, as a subjective part of this concept, refers to the recipient’s perceptions regarding how existing support is made available to satisfy their needs [21, 22].

Several studies have demonstrated that in both Western and Eastern communities, perceived social support is positively associated with HRQoL in certain groups, e.g. those with acute or chronic diseases [23–31], elderly populations [32, 33], immigrant workers and employees [34, 35]; however, few studies have assessed this relationship in general population and between genders [6, 36]. In this regard a community-based study conducted on a large population of American adults showed that compared to women, men reported better HRQoL as well as higher level of social support; however, there were no significant differences in the association between these two concepts between genders [36]. On the contrary, another study revealed a higher predictive power of social support for women’s QoL than for men, in an Italian population [6]. In addition, sex, age, educational level and job status were among the main socio-demographic indices which could improve physical or mental aspects of HRQoL [31].

HRQoL and social support as cultural and value-based concepts have been separately addressed only in a few Iranian studies. Those investigations that aimed to investigate the relation of these concepts in Iranian population have been focused on the particular employees, patients on hemodialysis, and those with coronary heart disorders and HIV [35, 37–40]. In this regard, considering the glaring lack of data on this relationship among general Iranian populations, this study assessed the hypothesized model which examined the network of associations among socio-demographic variables, social support and HRQoL in Iranian men and women by structural equation modeling.

## Methods

The population of this study were adults (> 19 years), participants of the Tehran Lipid and Glucose Study (TLGS), a community-based study designed and conducted among residents of district No. 13 of Tehran, aimed at determining the risk factors and prevention of non-communicable diseases [41]. Of TLGS participants who participated during 2015–2016 (*n* = 1139), after excluding outliers (*n* = 31) and missing data (*n* = 72), all adults participants who had complete data on Short-Form 12-Item Health Survey version 2 (SF-12v2) and Multidimensional Scale of Perceived Social Support (MSPSS) were recruited for current study (*n* = 1036). Prior to data collection, the ethics committee of the Research Institute for Endocrine Sciences (RIES) of Shahid Beheshti University of Medical Sciences approved the study and all participants signed an informed consent form.

Socio-demographic information (age, marital status, employment status and level of education) and data on perceived social support and health-related quality of life of participants were collected by trained interviewers, using standard questionnaires. Having chronic diseases was defined as diagnosed cancer, chronic kidney diseases, diabetes, hypertension and history of cardiovascular diseases. Perceived social support was assessed using the MSPSS developed by Zimet et al.. The MSPSS encompasses 12 items and three subscales. Each subscale includes four items and assesses perceived social support from three different sources including family, friends and significant others. For scoring each item, a seven-point scale ranging from 1 (very strongly disagree) to 7 (very strongly agree) was used. The minimum and maximum total scores for the scale are 12 and 84 respectively and a higher total score indicates higher perceived social support. In the current study, the Iranian version of MSPSS was used; its validity and reliability have been reported in a previous study [42].

HRQoL was assessed using the SF-12v2, which is a generic measure of perceived health status, consisting of 12 items and eight subscales including physical functioning, role physical, bodily pain, general health, vitality, social functioning, role emotional and mental health. The subscale scores ranged from 0 to 100, indicating the lowest and highest level of health measured by the scale. Physical component summary (PCS) and mental component summary (MCS) scores were calculated using the appropriate scoring algorithms. Validity and reliability of the Iranian version of SF-12v2 has been reported previously [43].

### Statistical analysis

**Fig. 1:**
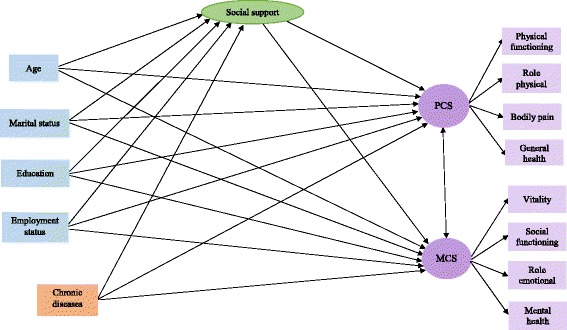
Conceptual model for the association between socio-demographic factors, social support health-related quality of life (HRQoL)

For continuous variables, mean ± SD and for categorical ones, frequency (percent) were reported as descriptive statistics. Means of continuous and the distribution of categorical variables were compared between genders using the Independent samples t-test and the Chi-Square test respectively. Associations between socio-demographic, social support and quality of life scales were examined using Structural Equation Modeling (SEM) [44]. As shown in the conceptual frame work of the inter-relationship between variables (Fig. 1), social support and HRQoL were considered as latent constructs and social support was considered as mediator in the relationship between socio-demographic and HRQoL. To test the hypothesized model across gender groups and compare them, SEM multiple-group analysis was applied. In the first model (unconstrained model), all parameters were considered different in men and women. In the multiple group modeling, some constraints about parameters equality between men and women were considered. Constrained models were defined as follows: *Measurement weights model:* Equal factor loadings for measurement model of social support and quality of life constructs in men and women; *Structural weights model:* Equal factor loadings and regression weights between latent variables in men and women; *Structural covariance model:* Equal covariance for latent constructs in men and women; *Structural residuals model:* Equal residual variances for latent constructs in men and women and the *measurement residuals model:* All parameters were considered equal in men and women. Fit indices of SEM models after modifying were calculated and compared to their acceptable thresholds [45]. Statistical analysis and computations were done by IBM SPSS Statistics & AMOS version 22.

## Results

Of 1036 participants, 40.9% were male and mean ages were 50.3 ± 16.3 and 49.6 ± 14.0 years in men and women respectively. Descriptive statistics for socio-demographic variables, perceived social support and HRQoL scores are presented in Table 1. Mean age and distribution of marital status did not differ significantly in men and women; however, there were significant differences in distributions of level of education and employment status. Most men and women had secondary education and a higher percentage of men (34.0%) had higher education compared to women (29.1%). In terms of employment status, majority of women were housewives (70.9%) and majority of men were employed (66.3%). About half of both men (42.5%) and women (50.7%) had chronic diseases with significantly higher prevalence in women compared to men (*p* < 0.05). In terms of social support scores, except for social family subscale scores (*p* = 0.003), there were no significant differences between men and women. However, as indicated in Table 1, in all subscales of HRQoL, men had significantly higher HRQoL scores compared to women (*p* < 0.001).

**Table 1:** Descriptive statistics of study participants

	Male (*n* = 424)	Female (*n* = 612)	*P* value
Age (years)	50.3 ± 16.3	49.6 ± 14.0	0.52
Marital status n(%)			
-Single	83 (19.6)	139 (22.7)	0.257
-Married	341 (80.4)	473 (77.3)	
Level of education n(%)			
-Primary	109 (25.7)	208 (34.0)	0.016
-Secondary	171 (40.3)	226 (36.9)	
-Higher	144 (34.0)	178 (29.1)	
Employment status n(%)			
- Unemployed/student/housewife	32 (7.5)	434 (70.9)	< 0.001
- Unemployed, but had other sources of income	111 (26.2)	71 (11.6)	
- Employed	281 (66.3)	107 (17.5)	
Chronic diseases			
-No	244 (57.5)	302 (49.3)	0.011
-Yes	180 (42.5)	310 (50.7)	
Social support scores	65.8 ± 12.2	64.6 ± 12.6	0.15
- Family	24.0 ± 4.3	23.2 ± 4.8	0.003
- Friend	19.1 ± 6.0	19.2 ± 6.2	0.88
-Significant other	22.6 ± 5.3	22.3 ± 5.7	0.32
SF-12 scores			
-Physical Function	87.2 ± 22.9	80.6 ± 25.2	< 0.001
-Role Physical	84.6 ± 20.5	73.3 ± 24.1	< 0.001
-Bodily pain	85.1 ± 21.0	75.4 ± 24.7	< 0.001
-General Health	49.9 ± 22.2	45.2 ± 22.5	< 0.001
PCS	49.6 ± 7.3	47.2 ± 8.5	< 0.001
-Vitality	69.2 ± 24.4	60.5 ± 25.9	< 0.001
-Social Function	84.3 ± 24.9	77.5 ± 27.1	< 0.001
-Role Emotional	80.3 ± 22.2	71.0 ± 24.2	< 0.001
-Mental Health	74.8 ± 20.3	65.7 ± 22.1	< 0.001
MCS	50.6 ± 9.6	46.5 ± 11.1	< 0.001

Continuous variables are represented as mean ± SD and the categorical ones are presented as frequency (percentage)

*PCS* Physical component summary, *MCS* Mental component summary

The results of model comparison and fit indices of structural model considering different constraints are presented in Table 2. All evaluations about associations and the conceptual frame work of the inter-relationship between variables are reported based on the unconstrained model. In the unconstrained model (all parameters were considered different in men and women) we achieved acceptable fit indices and compared to one of constrained models entitled “measurement weights” (equal factor loadings allowed for measurement models of social support and quality of life constructs in men and women), no statistical difference was observed between two models (∆χ^2^ = 12.96, DF = 8, *P* = 0.11). All other constrained models were statistically different from the unconstrained one and the model with different parameters between men and women had better fit indices.

**Table 2:** Model comparison, fit indices and results of chi-square test for comparisons between the two models

Model	DF	χ^2^/DF	RMSEA	SRMR	CFI	GFI	NFI	IFI	AIC	Model comparisons(χ^2^,DF)
Unconstrained^a^	158	3.82	0.052	0.079	0.91	0.93	0.88	0.91	831.5	Assuming to be correct
Measurement weights^b^	166	3.71	0.051	0.080	0.91	0.93	0.88	0.91	828.5	12.96, DF = 8
Structural weights^c^	183	3.53	0.050	0.083	0.91	0.92	0.87	0.91	823.5	41.94*, DF = 25
Structural covariance^d^	196	5.30	0.064	0.096	0.83	0.90	0.80	0.83	1190.5	434.9**, DF = 38
Structural residuals^e^	200	5.28	0.064	0.098	0.83	0.89	0.80	0.83	1200.2	452.7**, DF = 42
Measurement residuals^f^	215	5.10	0.063	0.098	0.82	0.89	0.79	0.82	1209.9	492.5**, DF = 57

Unconstrained model assuming to be correct, other proposed models ^b-f^ were compared to unconstrained model using chi-square difference test

*DF* degree of freedom, *RMSEA* root of mean square error approximation, *SRMR* standardized root mean square residual, *CFI* comparative fit index, *GFI* goodness of fit index, *NFI* normed fit index, *IFI* incremental fit index, *AIC* Akaike information criterion

*p < 0.05, **p < 0.001. ^a^All of the parameters were considered different in men and women, ^b^Equal factor loadings for measurement model of social support and quality of life constructs in men and women, ^**c**^Equal factor loadings and regression weights between latent variables in men and women, ^d^Equal covariance for latent constructs in men and women, ^e^Equal residual variances for latent constructs in men and women ^f^All parameters were considered equal in men and women

**Fig. 2:**
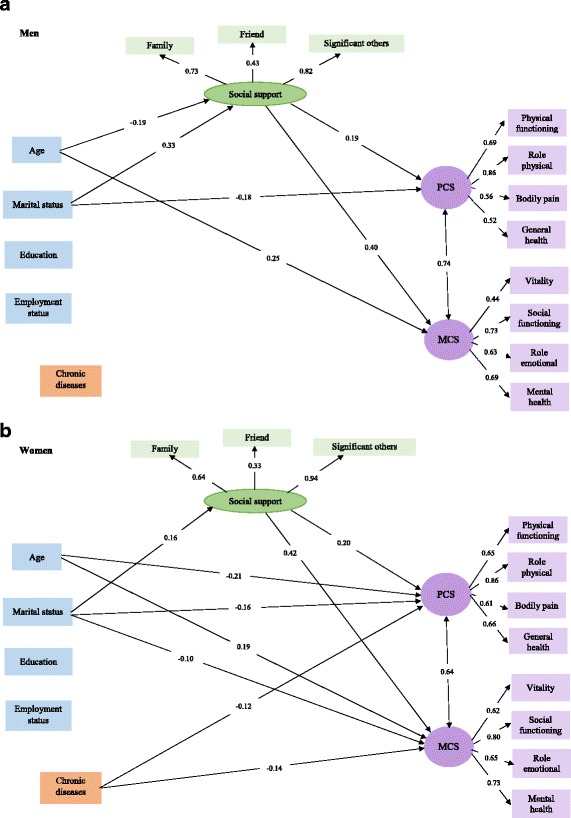
Final structural models after testing the association between socio-demographic factors, social support and health-related quality of life (HRQoL) (**a** Men and **b** Women)

All hypothesized associations in the conceptual model among socio-demographic variables, perceived social support and HRQoL are demonstrated in Fig. 1; perceived social support, PSC and MCS were considered as latent constructs in the model. Figure 2 indicates structural models after testing the association between socio-demographic factors, social support and HRQoL by gender. Fit indices for SEM in men (χ^2^ = 298.2, df = 79, χ^2^/df = 3.77, CFI = 0.88, GFI = 0.92, RMSEA = 0.08, SRMR = 0.08) and women (χ^2^ = 300.7, df = 79, χ^2^/df = 3.80, CFI = 0.92, GFI = 0.94, RMSEA = 0.07, SRMR = 0.06) display acceptable fit for hypothesized models in gender groups. Only significant associations and their corresponding coefficients (β) are drawn in Fig. 2. The findings of structural equations modeling analysis are summarized as follows: In terms of social support, being married in both men (β = 0.33; *p* < 0.001) and women (β = 0.16; *p* < 0.001) and lower age, only in men (β = − 0.19; *p* < 0.05) were significantly associated with higher level of perceived social support. PCS and MCS were significantly correlated in both men (*r* = 0.74; *p* < 0.001) and women (*r* = 0.63; *p* < 0.001). In terms of PCS, being single and higher perceived social support in both genders and lower age and not having any chronic diseases only in women, were significantly associated with higher physical HRQoL scores. In terms of MCS, higher age and higher perceived social support were significantly associated with better mental HRQoL scores in both genders; however, in women, being single and not having chronic diseases were also significantly associated with better mental HRQoL scores.

The effect differences between men and women were tested using multi-group analysis. Standardized coefficients and their corresponding critical ratios (CR) for each gender are reported in Table 3. Findings indicate that the effect of age on physical HRQoL and the effects of chronic diseases and social support on mental HRQoL were significantly different between men and women with higher effects in women, compared to men.

**Table 3:** Results of the structural equation modeling analysis: gender-specific relationships between socio-demographic factors, social support and HRQoL

		Male		Female		Difference CR
		Coefficient β	CR	Coefficient β	CR	
Age	Social support	−0.191	−2.28*	−0.041	−0.65	1.32
Marital status^a^		0.326	4.13**	0.164	3.37**	−1.84
Education^b^		−0.006	−0.10	0.066	1.16	0.87
Employment status^c^		− 0.070	−1.19	− 0.017	− 0.33	0.80
Chronic diseases^d^		0.041	0.64	0.079	1.44	0.43
Age	PCS	− 0.034	−0.44	− 0.216	−3.70**	− 2.16*
Marital status		− 0.179	− 2.43*	−0.164	−3.75**	0.15
Education		0.081	1.48	0.077	1.47	−0.02
Employment status		0.063	1.17	−0.019	−0.41	− 1.19
Chronic diseases		−0.088	−1.49	− 0.122	− 2.43*	− 0.46
Social support		0.193	3.15**	0.201	4.54**	0.26
Age	MCS	0.245	2.82**	0.185	2.95**	0.53
Marital status		−0.067	−0.87	− 0.096	− 2.06*	− 0.71
Education		−0.050	−0.86	0.083	1.47	1.70
Employment status		0.027	0.47	−0.026	− 0.52	−0.70
Chronic diseases		−0.004	−0.06	− 0.144	− 2.64**	− 2.04*
Social support		0.397	4.90**	0.422	7.54**	2.09*
PCS^e^		0.744	6.05**	0.634	8.26**	1.18

*PCS* Physical component summary, *MCS* Mental component summary

*p < 0.05, **p < 0.001, ^a^Single group was considered as reference group, ^b^Higher education was considered as reference group, ^c^Unemployed group was considered as reference group, ^d^Not having chronic diseases was considered as reference group, ^e^correlation coefficient

## Discussion

The present study aimed at testing a conceptual model of associations among socio-demographic factors, perceived social support and health-related quality of life (HRQoL) in an urban Iranian population. Based on our findings, marital status and social support in both genders and age and having chronic diseases only in women were factors directly associated with the physical aspect of HRQoL. Furthermore, age and social support in both genders and marital status and having chronic diseases only in women, were factors directly associated with the mental aspect of HRQoL. These findings highlight the prominent role of perceived social support in both aspects of perceived health in Iranian adults.

In the current study, perceived social support from family and significant others were higher than friends. Recent findings imply that, compared to friends, family members and significant others are more important sources of perceived social support in our society. Moreover, current findings indicate no significant differences in perceived social support scores, except for social family subscale scores which were significantly higher in men, compared to women. Another study conducted among Tehranian medical personnel reported no gender differences in social support [35]. In terms of socio-demographic factors associated with perceived social support, marital status was significantly associated with perceived social support in both genders. Findings of higher perceived social support in Iranian married men and women, compared to their single counterparts are in line with other previous studies [46–48]. Furthermore, younger men perceived higher social support, compared to older men, implying that with increasing age, the ability of men to make social connections decreases.

In the current study, social support was significantly associated with both aspects of HRQoL in both genders, a finding in agreement with previous studies from different countries [7, 36]; other studies conducted on different Iranian populations [35, 49] also found that perceived social support was an important correlate of HRQoL; these findings indicate that we/us humans are “social beings” and having good social relations and strong social ties can influence both the physical and mental aspects of health. An interesting finding of the current study was the different gender specific effect of social support on the mental aspect of HRQoL which was significantly higher in women compared to men. Another study conducted in Iran also revealed that insufficient perceived social support was shown to be associated with postpartum depression disorder in Iranian women [50], findings emphasizing the importance of perceived social support on mental aspect of health in women.

Among socio-demographic factors, the current conceptual model indicated that only age and marital status were significantly and directly associated with HRQoL and socio-economic factors (assessed by level of education and employment in this study) were not significantly associated with HRQoL in both genders emphasizing the important roles of marital status and age, compared to socio-economic status in perception of health among Iranian adults. Although married individuals were consistently found to have better perception regarding their health status in previous studies [51, 52], our findings indicate that single individuals reported better HRQoL scores as single women reported higher HRQoL scores in both physical and mental HRQoL and men reported higher scores only in physical HRQoL. Current evidence indicates that perceived social support was found to be a mediator of this association [53], data in line with our findings. Another important and interesting finding of this study was that while previous studies consistently reported poorer HRQoL in individuals suffering from different chronic diseases [54–57]; based on the current conceptual model, no significant association was found between chronic diseases and HRQoL in men. However, in women, chronic diseases were associated with poorer HRQoL in both the physical and mental aspects indicating different patterns of association among HRQoL, perceived social support and other related factors in Iranian men and women.

In addition, based on gender specific analysis, factors including age, having chronic diseases and social support had significantly stronger effects on HRQoL in women, compared to men, implying more important roles for social factors in perception of health in women, compared to men.

Few studies in Iran have investigated the associations between perceived social support and HRQoL and those that did focused mainly on *specific groups*. The current study is among the first efforts that reports the associations among socio-demographic factors, perceived social support and health-related quality of life (HRQoL) in a general urban Iranian population with different socio-economic statuses. In interpreting the findings of this study, the following limitations should be considered; first, causality cannot be assumed from findings of this study due to its cross-sectional nature. More robust research designs, such as prospective cohorts or stepped wedge clusters are recommended to try and gauge causal relationships. Second, the participants of this study were limited to Tehranian adults and the results are not generalizable to other parts of Iran specifically rural areas; therefore, to consider these findings for policy making, conducting further studies in rural areas and other cities of Iran is definitely recommended. Moreover, in the current study, paired structure of data for couple participants was not considered which could affect the current results. To tackle this limitation, correlated data models such as random effects SEM recommended to be applied in future studies with large number of pairs or couples. Finally, as some other potential factors associated with HRQoL such as income level and gender-specific psycho-social conditions and opportunities were not included in the model, assessing these related factors is also recommended in future studies in this field.

## Conclusion

In conclusion, among social factors considered in this study, age, marital status and perceived social support were significant determinants of both physical and mental HRQoL. Additionally, chronic diseases were associated with HRQoL only in women. The current structural model provide beneficial information for planning future health promotion programs aiming at improving HRQoL among Tehranian adults. Considering the nature of other significant social determinants of HRQoL, only social support can be included in intervention programs. Therefore, designing interventions aimed at helping individuals to foster their social network and make better social ties, especially with their family members are recommended.

## Abbreviations

HRQoL: Health-related quality of life
MCS: Mental component summary
MSPSS: Multidimensional Scale of Perceived Social Support
PCS: Physical component summary
SEM: Structural Equation Modeling
SF-12v2: Short-Form 12-Item Health Survey version 2
TLGS: Tehran Lipid and Glucose Study
